# Relationships between Circulating Biomarkers and Body Composition Parameters in Patients with Metabolic Syndrome: A Community-Based Study

**DOI:** 10.3390/ijms25020881

**Published:** 2024-01-10

**Authors:** Nader Tarabeih, Alexander Kalinkovich, Shai Ashkenazi, Stacey S. Cherny, Adel Shalata, Gregory Livshits

**Affiliations:** 1 Department of Morphological Sciences, Adelson School of Medicine, Ariel University, Ariel 40700, Israel; nader@ariel.ac.il (N.T.); shaias@ariel.ac.il (S.A.); 2 Department of Anatomy and Anthropology, Faculty of Medicine, Tel-Aviv University, Tel-Aviv 69978, Israel; alexander.kalinkovich@gmail.com (A.K.); cherny@tauex.tau.ac.il (S.S.C.); 3 The Simon Winter Institute for Human Genetics, Bnai Zion Medical Center, The Ruth and Bruce Rappaport Faculty of Medicine, Technion-Israel Institute of Technology, Haifa 32000, Israel; adel.shalata@gmail.com

**Keywords:** metabolic syndrome (MetS), adipokines, body composition, monocytes, inflammation

## Abstract

Metabolic syndrome (MetS) is a complex disease involving multiple physiological, biochemical, and metabolic abnormalities. The search for reliable biomarkers may help to better elucidate its pathogenesis and develop new preventive and therapeutic strategies. In the present population-based study, we looked for biomarkers of MetS among obesity- and inflammation-related circulating factors and body composition parameters in 1079 individuals (with age range between 18 and 80) belonging to an ethnically homogeneous population. Plasma levels of soluble markers were measured by using ELISA. Body composition parameters were assessed using bioimpedance analysis (BIA). Statistical analysis, including mixed-effects regression, with MetS as a dependent variable, revealed that the most significant independent variables were mainly adipose tissue-related phenotypes, including fat mass/weight (FM/WT) [OR (95% CI)], 2.77 (2.01–3.81); leptin/adiponectin ratio (L/A ratio), 1.50 (1.23–1.83); growth and differentiation factor 15 (GDF-15) levels, 1.32 (1.08–1.62); inflammatory markers, specifically monocyte to high-density lipoprotein cholesterol ratio (MHR), 2.53 (2.00–3.15), and a few others. Additive Bayesian network modeling suggests that age, sex, MHR, and FM/WT are directly associated with MetS and probably affect its manifestation. Additionally, MetS may be causing the GDF-15 and L/A ratio. Our novel findings suggest the existence of complex, age-related, and possibly hierarchical relationships between MetS and factors associated with obesity.

## 1. Introduction

Metabolic syndrome (MetS) is a complex disorder that is considered a public health burden worldwide [1,2], leading to an increased risk of cardiovascular diseases (CVDs) [3,4], increased serum lipids, and type 2 diabetes mellitus (T2DM) [5]. MetS is a consequence of physiological, biochemical, and metabolic abnormalities, including simultaneous presence of abdominal obesity, insulin resistance, elevated arterial blood pressure, elevated triglycerides, and decreased high-density lipoprotein cholesterol (HDL-C) [6,7]. However, there is still a debate whether these elements of MetS themselves constitute separate pathologies or fall under a common, broader pathogenic process [3,6,8].

There are several hypothetical mechanisms underlying the pathophysiology of MetS, with insulin resistance [9,10] and chronic inflammation, particularly adipose tissue (AT) inflammation [11,12], being the most common. In this regard, growth and differentiation factor 15 (GDF-15), a member of the transforming growth factor-β (TGF-β) superfamily [13], has been linked with several MetS pathologies, including T2DM, obesity, hypertension, and CVDs [14,15]. Elevated circulating levels of GDF-15 were found in MetS patients [16,17,18]. GDF-15 is expressed in AT and secreted from adipocytes [19], suggesting that GDF-15 also acts as an adipokine.

It is well established that adipokines, mostly leptin and adiponectin, and the ratio leptin/adiponectin (L/A), are associated significantly with the development of obesity and insulin resistance [20,21,22]. Of interest, the association of T2DM risk with the L/A ratio is stronger than with leptin or adiponectin alone [23]. Of interest also is that a higher GDF-15/adiponectin ratio (G/A) was found to be independently associated with an increased risk of T2DM, suggesting that this ratio may serve as a biomarker of T2DM [24].

Increased circulating levels of several other adipokines, specifically chemerin and adipsin, as well as natural glycoprotein follistatin, are associated with MetS-related conditions [25,26,27,28,29] and are also involved in inflammation [30,31,32,33]. It has been shown that elevated circulating levels of chemerin correlate with insulin resistance and inflammation in patients diagnosed with MetS [34]. Adipsin plays an important role in glucose and lipid metabolism, energy balance, and maintenance of islet β-cell function, and its plasma concentrations positively correlate with metabolic risk abnormalities in nonalcoholic fatty liver disease [35]. Recently, it has been reported that plasma levels of follistatin are associated with an increased risk of T2DM by inducing adipose tissue insulin resistance [26]. Moreover, in a further study, these authors observed follistatin levels to be associated with an increased risk of heart failure, which may be mediated in part by T2DM, but are also independently associated with stroke, ischemic stroke, and other pathological conditions [36].

There is a substantial bulk of evidence indicating that systemic inflammation is also involved in the development of MetS [37]. One of the common systemic inflammatory markers, which has been used for the assessment of inflammation in CVDs, is monocyte to high-density lipoprotein cholesterol ratio (MHR) [38,39,40]. It was found that MHR was increased in MetS patients compared to controls and correlated positively with the severity of MetS [41]. We therefore wanted to test the extent to which this inflammatory marker is associated with MetS in our sample.

Accumulating evidence suggests that changes in body composition are associated with changes in metabolic parameters, which in turn increase the risk of MetS [42,43,44]. For example, lean body mass and appendicular skeletal mass demonstrate a protective effect on MetS risk, whereas fat mass (FM) increases MetS risk [45]. However, it should be mentioned that published data on the possible association between body composition, in particular body mass index (BMI), and MetS, are still limited and controversial [45,46,47,48,49,50]. Obesity measures, especially BMI, have been consistently linked with MetS [51], and some studies suggest that BMI is the most effective measure of body composition association with MetS [50]. However, other studies have concluded that an increased BMI does not necessarily increase the risk of MetS [52]. Moreover, because BMI is a surrogate measure of body composition including all types of tissues, in particular, fat, lean, and skeletal mass, testing specific compartments such as fat and muscular mass separately may more accurately assess the body composition association with MetS.

In summary, several previous studies examined the contribution of a variety of potential risk factors in the manifestation of MetS. This, however, is the first study, to the best of our knowledge, that evaluates the combined association of several inflammatory factors and several specific body composition characteristics with MetS.

## 2. Results

### 2.1. Sample Characteristics

Table 1 summarizes the mean values of the variables in the study population, separated by sex. The sample comprised 490 men and 589 women. No age differences were found (42.8 ± 0.62 vs. 43.2 ± 0.56 years, *p* > 0.05). The prevalence of MetS was 30% (325/1079), with no significant difference between women and men (31% (183/589) vs. 29% (142/490), respectively, *p* > 0.05 by using a *t*-test). As expected, body composition variables related to adipose tissue mass (BMI, FM/WT, and ECW/ICW ratio) were significantly higher in women than in men, while waist circumference, WHR, SMM/WT, and TBW were higher in men. Regarding the lipid profile, triglyceride levels were significantly higher in men, while the HDL-C/TC ratio was higher in women. Monocyte count, MHR, GDF-15 plasma levels, and G/A ratio were significantly higher in men, while leptin and adiponectin circulating levels, and L/A ratio were higher in women. There was no difference between men and women concerning other variables.

The body composition and the anthropometric variables were all significantly inter-correlated, similarly in both sexes (Table S1). The presented correlations were computed after adjusting for age for all the variables separately. To avoid redundancy and collinearity in further analysis of the data, only variables showing the most significant univariate correlations with MetS status were selected.

### 2.2. MetS Associations

Table 2 shows that patients with MetS were significantly older than those unaffected (51.89 ± 0.67 vs. 39.22 ± 0.46 years, *p* = 0.001). In addition, patients with MetS had significantly higher obesity features, specifically waist circumference, WHR, BMI, FM/WT, as well as ECW/ICW, than those without MetS, while SMM/WT was significantly lower in patients with MetS. However, it should be underlined that all these differences remained significant after adjustment of the study variables for age and sex.

The most relevant and interesting results were related to the comparison of circulating factors. Plasma levels of all circulating factors tested, except adiponectin, were significantly elevated in the MetS group (Table 2). For example, GDF-15 levels were 644.05 ± 23.18 pg/mL vs. 421.33 ± 8.80 pg/mL, respectively, and the difference remained highly significant after adjustment for age and sex (*p* = 1.14 × 10^−8^). As expected, adiponectin levels were decreased in obese individuals. In contrast, MHR, G/A, and L/A ratios were significantly higher in MetS patients (*p* < 0.0001) before and after adjustment for age and sex.

### 2.3. Multivariable Analysis

All potential predictor variables (covariates) that were significantly associated with MetS status in the univariate context (Table 2) were analyzed using mixed-effects logistic regression to examine the combined and independent effects of the covariates. As our sample consisted of nuclear and more complex families, appropriate regressions were used to account for family size and structure (Table 3).

The results show that FM/WT, ECW/ICW ratio, MHR, plasma GDF-15 levels, and L/A ratio were independently and significantly associated with MetS when adjustment for age and sex effects was not conducted and remained significant while controlling for age and sex (Table 3). The calculated odds ratios (OR, 95% CI) ranged from 0.79 (0.66–0.96) for ECW/ICW levels to 2.77 (2.01–3.81) for FM/WT.

Other parameters tested, which were significantly elevated in patients with MetS compared with those without (Table 2), were not retained in the final regression equation as independently associated covariates.

### 2.4. Additive Bayesian Network Analysis

**Figure 1:**
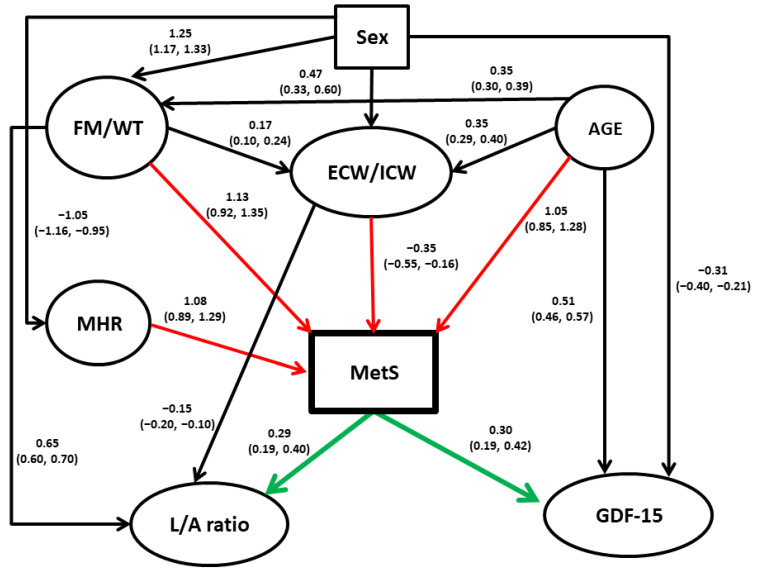
Directed acyclic graph among study measures. A continuous variable appears in ovals. All quantitative variables were standardized prior to analysis. Coefficients on the arcs (paths) between “parents” (independent variables) and “children” (dependent variables) are the modes (beta) obtained from the posterior distributions from the Bayesian modeling, with the corresponding 95% credible intervals presented below in parentheses. These coefficients are analogous to regression coefficients from multiple regression models. Red arrows denote direct influences on MetS, and green arrows indicate direct influences of MetS on other variables. Abbreviations: MetS, metabolic syndrome; FM/WT, fat mass/weight ratio; ECW/ICW, extracellular water/intracellular water ratio; MHR, monocyte to high-density lipoprotein cholesterol ratio; L/A ratio, leptin/adiponectin ratio.

This analysis was conducted to clarify relationships among the variables examined in this study, possibly of the causal form. The following variables were considered in the analysis: MetS, FM/WT, ECW/ICW ratio, MHR, GDF-15 levels, L/A ratio, age, and sex (Table 3). The procedure employed resulted in a need for up to four parents (causal variables) for each variable in the model, based on the likelihood not increasing with the addition of more parents, and a total of 16 arcs. The relationships uncovered among the variables can be seen in Figure 1, with the parameter estimates shown on the arcs and 95% credible intervals in brackets under them. The procedure standardizes continuous variables prior to the analysis.

As seen, most covariates in the study were significantly dependent on sex and age. The most important links were, however, the direct connections found between MHR, ECW/ICW, FM/WT, and age with MetS. Interestingly, the effects of FM/WT on MetS appear to be linked not only directly but also indirectly via ECW/ICW. The latter association, however, is comparatively small and negative. The results also suggest that MetS causes both GDF-15 levels and L/A ratio and not vice versa.

## 3. Discussion

The aims of the present study included a simultaneous analysis of the associations of selected inflammatory factors and the specific components of body composition with MetS and the possible network of the interconnections between the study variables. To our knowledge, no study to date has uncovered the possible direct (causal) and indirect relationships underlying the complex network of variables potentially affecting the risk of MetS.

Our additive Bayesian network modeling found that adipose tissue-related phenotypes, particularly FM/WT and an array of adipokines, appear to play a central role in the risk of MetS manifestation, while the latter appears to have a direct effect on GDF-15 and L/A ratio (Figure 1). It should be mentioned, however, that directions shown on the diagram should be taken with caution, and although they reflect the associations well, they should be tested and confirmed in the independent studies. It is of particular importance concerning GDF-15, the functions of which were recently summarized as “stress-, infection-, and inflammation-induced cytokine, which expression is increased in aging and suppresses immune responses” [53].

In addition, the network analysis points to the central role of aging in the variation of virtually all potential risk factors, albeit with differences between the sexes, in the manifestation of all phenotypes tested. This result agrees with a series of regression analyses (Table 2 and Table 3) that show that MetS is significantly and independently correlated with body composition parameters, particularly the markers of obesity (FM/WT and ECW/ICW ratio), the adipose tissue-related adipokine L/A ratio, the inflammation-related factors as assessed by using MHR, and the multifunctional aging biomarker GDF-15 [53].

Because the present findings suggest a significant and relatively strong possible direct effect of MHR and FM/WT ratio on MetS manifestation, and because MetS in turn significantly increases the risk of CVD and coronary heart disease (CHD) [4,54,55,56], these factors should be considered when the risk of CVD is evaluated.

MetS is a major global health problem that increases the risk of developing CVDs and T2DM [7]. It involves several complicated mechanisms that have not yet been fully deciphered [7] and remains a challenge due to its poorly understood etiology and pathogenesis as well as the lack of reliable mechanistic biomarkers. Once these biomarkers are validated, this will pave the way to clarify the pathogenic metabolic pathways underlying the development of MetS and its associated health complications. It may also help develop new preventive and therapeutic strategies.

It should be stressed that a statistical analysis of cross-sectional data reflects associations and is only suggestive of causality. However, if the reported findings prove to be true, they could have a significant impact on our understanding of the pathophysiology of MetS and the most effective treatment options. Some of the results we present are generally well supported by the published data. For example, it has been repeatedly reported that fat accumulation plays an important role in the development of MetS [57]. Our univariate analysis showed that ECW/ICW is significantly higher in MetS-diagnosed individuals than in those without MetS (Table 2), but its independent effect is minor and negative (Figure 1). However, ABN modeling also showed the possible effect of age and FM/WT on ECW/ICW. The latter agrees with data that an elevated ECW/ICW ratio is observed in obesity [58,59,60], T2DM [61], and hypertension [62]. Age and FM/WT also have much stronger possible direct effects on MetS, and it is likely that the negative association of ECW/ICW with MetS in multivariable analysis is due to its adjustment for age and FM/WT.

The role of adipokines, mainly leptin and adiponectin, in the pathogenesis of MetS, is well established. High leptin and decreased adiponectin levels, respectively, are associated with obesity, insulin resistance, T2DM, and CVDs [22,63,64]. Notably, the association of T2DM risk with the L/A ratio is stronger than with leptin or adiponectin alone [23,65], as is observed in the present analysis of MetS. These results suggest that the L/A ratio may be a useful indicator of insulin resistance and for assessing the effectiveness of antidiabetic therapy. In support, it has been reported that both the calculated HOMA-IR index and the L/A ratio can be used to identify insulin resistance in obese individuals [66].

Our study shows a consistently significant association between GDF-15 circulating levels and MetS score. GDF-15 blood levels were proposed to be a clinically relevant biomarker within the context of MetS, as they have been associated with several MetS components, such as hyperglycemia, hypertension, and hyperlipidemia [67,68,69,70,71], high waist circumference, WHR and CRP, and low HDL-C [18,72]. In animal models, GDF-15 showed anorectic activity, mainly through a reduction in food intake, suggesting that it may counteract the development of MetS, particularly through weight reduction [73]. It has also been shown that mice fed a high-fat diet and treated with monoclonal antibodies that inhibit GDF-15 have increased inflammation and white adipose tissue (WAT) volume [74], whereas the administration of recombinant GDF15 or genetic overexpression of GDF-15 is associated with decreased circulating cytokines and WAT inflammation levels [75]. Although these observations suggest that the anti-obesity activity of GDF-15 may be related to its anti-inflammatory effect, it remains to be seen whether the anti-inflammatory effect of GDF-15 on WAT is direct and independent of weight (e.g., through macrophages or other immune cells) or the effect of GDF-15 on low-grade inflammation is only indirect through weight loss [15]. The potential anti-inflammatory effect of GDF-15 is also associated with its possible protective effect against atherosclerosis [76]. A recent meta-analysis revealed that GDF-15 consistently adds prognostic information for myocardial infarction and stroke, CV death, and heart failure beyond clinical risk factors and cardiac biomarkers of CVDs [77]. The prognostic significance of elevated circulating GDF-15 levels has also been demonstrated for T2DM [78]. However, it remains unclear whether the effects of GDF-15 in MetS are causative or whether its levels are a consequence of the disease. Our network analysis indicates that the latter assumption could be possible, and MetS likely influences GDF-15 levels and not vice versa.

MHR is a novel systemic inflammatory marker that has been associated with major adverse cardiovascular characteristics [38,39,40] and T2DM [79]. Moreover, the predictive ability of MHR for clinical outcomes may be even better than independent monocyte counts and HDL-C concentration [80]. Monocytes and macrophages accumulate in inflamed AT and produce a wide range of pro-inflammatory molecules, which, in turn, exacerbate chronic inflammation associated with MetS-related CVDs and T2DM [81,82,83]. Notably, HDL-C has been shown to prevent inflammatory responses by acting directly on monocytes [84]. These data suggest the involvement of MHR in MetS and explain the increased MHR in patients with MetS in our study and others [85].

As with other studies, the present one has some limitations. The most important one is that it is a cross-sectional design, which means we cannot draw definitive conclusions about the causal relationship between MetS and the significantly associated factors. To better establish cause-and-effect relationships, as well as truly evaluate the predictive power of the specific factor(s), longitudinal studies are required. In addition, our study did not evaluate testosterone levels in male patients or the presence of polycystic ovary syndrome in women. These factors could be associated with the metabolic syndrome [86,87] and therefore may introduce some bias in the parameter estimates. Moreover, this research was performed in a single ethnically and culturally homogeneous population. Therefore, further studies should be conducted on other populations to ensure that the results can be generalized.

## 4. Materials and Methods

### 4.1. Study Design and Ethics

The data were collected from 1079 individuals (mean age 43.0 ± 13.8 years) enrolled in outpatient clinics in Sakhnin (Israel) from 2015 to 2022. All participants were from an ethnically and culturally homogeneous population of Israeli Arabs, being members of 98 nuclear and more complex three-generation families [88,89]. They provided complete medical records or consented to provide access to their medical records. The inclusion criterion for the study group was age of 18 to 78 years. The exclusion criteria were pregnancy, traumatic disorders, systemic inflammatory or autoimmune disorders, severe heart problems, neoplastic disease, and a history of malignancy. All participants in the study sample were assessed by certified and experienced nurses. Demographic data, anthropometrics, body composition measurements, and blood samples (30 cc) were collected from all individuals.

This research was approved by the IRB-Helsinki Committee (Number: 042/2013K, Date: 4 November 2013) of the Meir Medical Center, Kfar Saba, Israel and the Ethics Committee of Tel Aviv University, Tel Aviv, Israel. Written informed consent was obtained from all participants before their inclusion.

### 4.2. Definition of Metabolic Syndrome

According to the NCEP ATP III [90], MetS is defined as the presence of at least 3 of the following 5 components: (1) a waist circumference ≥ 102 cm in men and ≥88 cm in women; (2) serum triglycerides (TG) ≥ 150 mg/dL or drug treatment for hypertriglyceridemia; (3) serum HDL-C < 40 mg/dL in men or <50 mg/dL in women, or drug treatment for lowering low-density lipoprotein cholesterol (LDL-C); (4) a systolic blood pressure (SBP) ≥ 130, a diastolic blood pressure (DBP) ≥ 85 mmHg, or being on antihypertensive drug treatment; (5) fasting blood glucose ≥ 100 mg/dL or drug treatment of hyperglycemia.

### 4.3. Anthropometric and Body Composition Assessments

Demographic, anthropometric, and body composition data were collected and recently described in detail elsewhere [33,88]. They included height (cm), weight (kg), and waist and hip circumferences (cm), and calculated body mass index (BMI) in kg/m^2^ and waist-to-hip ratio (WHR). Body composition parameters were assessed by using body impedance analysis (BIA) using the BIA101 device (Akern Bioresearch, Pontassieve, Italy) [91], which is safe, reliable, simple, accurate, and inexpensive [91,92]. BIA analysis included evaluation of fat mass (FM), skeletal muscle mass (SMM) in kilograms, total body water (TBW), extracellular water (ECW), and intracellular water (ICW) in liters. ECW-to-ICW ratio (ECW/ICW) and TBW were chosen due to their fundamental physiological significance [93]. Because body mass components are interrelated and depend on body weight (WT), they were used as ratios to body weight, i.e., FM/WT and SMM/WT.

### 4.4. Blood Pressure Measurements

SBP and DBP were measured twice by a nurse, using a blood pressure monitor (BPM), in a sitting position, at a 15 min interval, and mean values were used for analysis.

### 4.5. Analysis of Soluble Markers

Venous blood samples were obtained by venipuncture following overnight fasting in all individuals in the sample. Within one hour after collection, samples underwent centrifugation at 1800× *g* for 15 min at 4 °C. Plasma fractions were separated and stored in aliquots at −80 °C. Quantities of soluble markers were detected by using ELISA using DuoSet kits (R&D systems, Minneapolis, MN, USA) according to the manufacturer’s protocols. The detection limits were as follows: 7.8 pg/mL for GDF-15, 46.9 pg/mL for follistatin, 16.7 pg/mL for chemerin, 31.2 pg/mL for leptin, 62.5 µg/mL for adiponectin, and 375 µg/mL for adipsin. The intra- and inter-assay coefficients of variation were between 2.3 and 8.6%. Fasting blood samples were obtained for complete blood counts, glucose, and lipid profile, including triglycerides, total cholesterol (TC), HDL-C, and LDL-C. MHR was obtained by dividing monocyte count (10^3^ cells/μL) by HDL-C levels (mg/dL) as previously described [80,94]. Due to the significant deviation of some variables from normality, the original measurements of GDF-15, leptin, and follistatin were subjected to log-normal transformation prior to analysis.

### 4.6. Statistical Analysis

Statistical analysis of the data was conducted using Statistica 64 (TIBCO Software, version 13.5, Palo Alto, CA, USA) and R (version 4.3.1) [95]. The first stage of the analysis included identification of major covariates (potential predictors) for MetS status (yes vs. no). *T*-tests, parametric, and non-parametric (Kruskal–Wallis) ANOVAs were conducted to compare continuous variables between the groups, followed by correlation/regression analysis.

Subsequently, the best potential predictors of MetS were simultaneously examined in logistic mixed-effects models, allowing for familial correlations based on the degree of kinship. We used the kinship2 package (version 1.9.6) [96] for R to generate kinship matrices within families for use with the relmatGlmer function in the lme4qtl R package (version 0.2.2) for binary dependent variables [97]. Prior to analysis, we imputed missing data using the R package mice (version 3.16.0) [98] with default options. This process was implemented at the final stage of the analysis of MetS to accurately establish possible relationships with contributing biochemical markers and body composition variables.

### 4.7. Additive Bayesian Network Modeling

Finally, to explore the underlying causal structure for the variables examined, we used additive Bayesian network models [99], as implemented in the R package ABN, version 3.0.1 [100,101], with JAGS software, version 4.3.0, to bootstrap data for correcting for overfitting [102]. ABN modeling is a data-driven, exploratory, statistical method for uncovering causation among a set of variables which are correlated and is ideally suited for hypothesis generation when there is little theoretical basis for predicting the causal structure. By effectively performing a search of all possible causal relationships linking a set of variables, provided they constitute a directed acyclic graph (DAG), where no causal loops are present, a model of the causal structure can be inferred without the need for making strong prior assumptions. This model shows which variables directly cause which other variables. These links are referred to as arcs in DAG terminology, and the parameter estimates corresponding to the arcs are analogous to coefficients in multiple logistic regression. While ABN modeling does not require any causal assumptions if there are strong theoretical reasons for making assumptions, they should be made, and they will aid in finding the best model. We therefore did not permit causal arcs that were theoretically nonsensical. Our restrictions were as follows: (1) Sex was not permitted to be caused by any other variable, and (2) age at testing could not be caused by any other variable except sex. Prior to analysis, we imputed missing data using the R package mice [98] with default options, because ABN requires complete data for analysis. We used a four-stage analysis pipeline to arrive at a final causal model that guards against overfitting, as previously described [103,104]. Because it is theoretically possible that different causal structures could produce the same likelihood of the data [105,106], we caution that there are equivalent models with causal direction reversed, though this is not of major concern due to the strong theoretical basis for the direction of some of the arcs.

## 5. Conclusions

This study reports several novel findings with a prognostic potential for MetS. These include the significant and independent association of elevated MHR, GDF-15 levels, and L/A ratio, as well as FM/WT. These data corroborate multifactorial molecular mechanisms involved in MetS pathogenesis and suggest that several combined biomarkers should be used to accurately predict the course of the disease. We believe that if the functional role of the identified biomarkers in MetS pathophysiology is elucidated, they may serve as plausible therapeutic targets for MetS patients.

## Figures and Tables

**Table 1:** Baseline characteristics of the study population according to sex.

Characteristics	Male, N = 490	Female, N = 589	*p*
Age (years)	42.76 ± 0.62	43.20 ± 0.56	NS
Waist circumference (cm)	96.22 ± 0.51	94.04 ± 0.60	0.007
WHR (cm/cm)	0.93 ± 0.003	0.87 ± 0.003	0.001
BMI (kg/m^2^)	27.53 ± 0.19	28.39 ± 0.23	0.005
FM/WT (kg/kg)	0.25 ± 0.002	0.36 ± 0.003	0.001
SMM/WT (kg/kg)	0.37 ± 0.002	0.27 ± 0.001	0.001
TBW (L)	45.28 ± 0.26	33.44 ± 0.16	0.001
ECW/ICW ratio	0.86 ± 0.007	0.99 ± 0.007	0.001
SBP (mmHg)	125.29 ± 0.62	119.71 ± 0.63	6.44 × 10^−10^
DBP (mmHg)	79.05 ± 0.34	76.56 ± 0.31	1.29 × 10^−7^
TC (mmol/L)	177.63 ± 1.93	178.61 ± 1.54	NS
Triglycerides (mmol/L)	149.32 ± 5.31	112.48 ± 3.14	6.35 × 10^−10^
HDL-C/TC (mmol/L)	0.23 ± 0.003	0.29 ± 0.003	0.001
Fasting plasma glucose (mmol/L)	94.28 ± 1.06	97.78 ± 1.53	NS
Monocyte (×10^9^/L)	0.49 ± 0.01	0.36 ± 0.007	0.001
MHR (×10^3^)	13.00 ± 0.36	7.67 ± 0.18	0.001
GDF-15 (pg/mL)	520.47 ± 14.69	460.73 ± 13.10	0.002
Follistatin (pg/mL)	612.26 ± 21.37	619.21 ± 23.16	NS
Chemerin (ng/mL)	88.41 ± 1.24	90.41 ± 1.17	NS
Leptin (ng/mL)	11.43 ± 0.47	33.60 ± 0.92	0.001
Adiponectin (µg/mL)	3.36 ± 0.06	4.48 ± 0.07	0.001
Adipsin (µg/mL)	1.31 ± 0.01	1.30 ± 0.02	NS
G/A ratio	182.98 ± 7.16	119.67 ± 4.22	9.33 × 10^−15^
L/A ratio	3.93 ± 0.18	8.76 ± 0.29	0.001

Data presented as mean ± standard error; N, sample size; WHR, waist/hip ratio; BMI, body mass index; FM/WT, fat mass/weight ratio; SMM/WT, skeletal muscle mass/weight ratio; TBW, total body water; ECW/ICW, extracellular water/intracellular water ratio; SBP, systolic blood pressure; DBP, diastolic blood pressure; TC, total cholesterol; HDL-C/TC, high-density lipoprotein cholesterol/total cholesterol; MHR, monocyte to high-density lipoprotein cholesterol ratio; G/A ratio, GDF-15/adiponectin ratio; L/A ratio, leptin/adiponectin ratio. *p* is the significance level achieved upon a comparison between sexes by using a *t*-test, NS, non-significant.

**Table 2:** The characteristics of study subjects according to metabolic status.

Variable	Without MetS (N = 754)	With MetS (N = 325)	*p*	p*
Age (years)	39.22 ± 0.46	51.89 ± 0.67	0.001	
Waist circumference (cm)	90.57 ± 0.42	105.28 ± 0.59	0.001	0.001
WHR (cm/cm)	0.88 ± 0.002	0.95 ± 0.004	0.001	1.11 × 10^−16^
BMI (kg/m^2^)	26.46 ± 0.16	31.67 ± 0.25	0.001	0.001
FM/WT (kg/kg)	0.29 ± 0.003	0.36 ± 0.004	0.001	1.11 × 10^−16^
SMM/WT (kg/kg)	0.33 ± 0.002	0.29 ± 0.003	0.001	2.86 × 10^−13^
TBW (L)	37.83 ± 0.26	41.19 + 0.45	4.39 × 10^−11^	0.001
ECW/ICW ratio (L)	0.91 ± 0.006	0.97 ± 0.01	0.000006	1.18 × 10^−13^
SBP (mmHg)	118.07 ± 0.46	131.92 ± 0.81	0.001	0.001
DBP (mmHg)	75.87 ± 0.26	81.92 ± 0.40	0.001	0.001
TC (mmol/L)	179.35 ± 1.46	177.24 ± 2.18	NS	NS
Triglycerides (mmol/L)	101.48 ± 2.37	179.50 ± 6.35	0.001	0.001
HDL-C/TC (mmol/L)	0.28 ± 0.003	0.23 ± 0.003	0.001	0.001
Fasting plasma glucose (mmol/L)	89.21 ± 0.60	112.32 ± 1.97	0.001	0.001
Monocyte (×10^9^/L)	0.54 ± 0.14	0.42 ± 0.008	NS	NS
MHR (×10^3^)	8.63 ± 0.16	11.27 ± 0.28	0.001	1.84 × 10^−12^
GDF-15 (pg/mL)	421.33 ± 8.80	644.05 ± 23.18	0.001	1.14 × 10^−8^
Follistatin (pg/mL)	586.56 ± 19.45	679.48 ± 27.04	0.006	0.05
Chemerin (ng/mL)	84.06 ± 0.93	103.16 ± 1.66	0.001	4.72 × 10^−10^
Leptin (ng/mL)	20.07 ± 0.64	31.72 ± 1.40	0.001	6.65 × 10^−12^
Adiponectin (µg/mL)	4.12 ± 0.06	3.60 ± 0.08	0.000007	0.000002
Adipsin (µg/mL)	1.25 ± 0.01	1.40 ± 0.02	0.00001	0.01
G/A ratio	124.71 ± 3.94	205.45 ± 9.50	0.001	3.19 × 10^−9^
L/A ratio	5.29 ± 0.19	9.57 ± 0.41	0.001	2.22 × 10^−16^

Data presented as mean ± standard error; N, sample size; WHR, waist/hip ratio; BMI, body mass index; FM/WT, fat mass/weight ratio; SMM/WT, skeletal muscle mass/weight ratio; TBW, total body water; ECW/ICW, extracellular water/intracellular water ratio; SBP, systolic blood pressure; DBP, diastolic blood pressure; TC, total cholesterol; HDL-C/TC, high-density lipoprotein cholesterol/total cholesterol; MHR, monocyte to high-density lipoprotein cholesterol ratio; G/A ratio, GDF-15/adiponectin ratio; L/A ratio, leptin/adiponectin ratio. *p* is the significance level achieved upon a comparison of each MetS with the control group by using a *t*-test; NS, non-significant; p* is the *p*-value obtained when controlling for sex and age.

**Table 3:** Mixed-effects multivariate logistic regression analysis to explore the relationships between covariates and MetS (affected vs. unaffected).

MetS Status (325 Affected vs. 754 Controls)			
Independent Covariate	OR (95% CI)	Β (SE)	*p*
Age (years)	2.55 (2.00–3.26)	0.93 (0.12)	4.21 × 10^−14^
FM/WT (kg/kg)	2.77 (2.01–3.81)	1.01 (0.16)	3.75 × 10^−10^
ECW/ICW (L/L)	0.79 (0.66–0.96)	−0.22(0.09)	0.01
MHR (×10^3^)	2.53 (2.00–3.15)	0.93 (0.11)	4.30 × 10^−16^
GDF-15 (pg/mL)	1.32(1.08–1.62)	0.28 (0.10)	0.005
L/A ratio	1.50 (1.23–1.83)	0.40 (0.11)	0.00005

Data reported as odds ratio with 95% confidence intervals (OR (95% CI)), with corresponding beta coefficient and standard error (B (SE)); FM/WT, fat mass/weight ratio; ECW/ICW, extracellular water/intracellular water ratio; MHR, monocyte to high-density lipoprotein cholesterol ratio; L/A ratio, leptin/adiponectin ratio. At the initial stage of the present analysis, the following independent variables were tested in stepwise-forward manners: Age, sex, FM/WT, ECW/ICW, TBW, MHR, GDF-15, chemerin, and L/A ratio. All the variables in the analysis were standardized prior to statistical analysis. Only statistically significant terms are shown.

## Data Availability

Data are contained within the article and Supplementary Materials.

## Supplementary Materials

The following supporting information can be downloaded at https://www.mdpi.com/article/10.3390/ijms25020881/s1.
