# Supplementary material for: Relationships between Circulating Biomarkers and Body Composition Parameters in Patients with Metabolic Syndrome: A Community-Based Study
Source: Int J Mol Sci. 2024 Jan 10;25(2):881. doi: 10.3390/ijms25020881 (PMC10815336; doi:10.3390/ijms25020881)
Supplement: Supplementary file 1 [file ijms-25-00881-s001.zip › ijms-2785747-supplementary.pdf]

Table S1. Pearson correlations between body composition parameters and plasma levels of soluble markers in the study sample, in men and women separately; male correlations are shown above the diagonal and female below. All the variables were adjusted for age prior the analysis.

	**BMI**	**WHR**	**FM/WT**	**SMM/WT**	**ECW/ICW**	**GDF-15**	**Chemerin**	**Adiponectin**	**Adipsin**	**Follistatin**	**Leptin**	**L/A ratio**	**MHR**
**BMI**		0.51[2]	0.76[2]	-0.77[2]	0.01[n]	0.06[n]	0.31[2]	-0.17[2]	0.13[2]	0.08[n]	0.67[2]	0.58[2]	0.17[2]
**WHR**	0.27[2]		0.46[2]	-0.45[2]	0.30[2]	0.05[n]	0.20[2]	-0.20[2]	0.01[n]	0.07[n]	0.41[2]	0.37[2]	0.15[2]
**FM/WT**	0.80[2]	0.23[2]		-0.96[2]	0.26[2]	0.14[2]	0.30[2]	-0.09[1]	0.11[1]	0.04[n]	0.68[2]	0.52[2]	0.18[2]
**SMM/WT**	-0.67[2]	-0.21[2]	-0.93[2]		-0.20[2]	-0.12[1]	-0.29[2]	0.13[2]	-0.08[n]	-0.05[n]	-0.69[2]	-0.50[2]	-0.18[2]
**ECW/ICW**	0.15[2]	0.12[2]	0.29[2]	-0.23[2]		0.33[2]	0.16[2]	0.20[2]	0.15[2]	0.03[n]	0.07[n]	0.03[n]	0.07[n]
**GDF-15**	0.11[1]	0.12[2]	0.13[2]	-0.09[1]	0.23[2]		0.22[2]	-0.03[n]	0.15[2]	0.05[n]	0.09[1]	0.14[2]	0.15[2]
**Chemerin**	0.28[2]	0.12[2]	0.28[2]	-0.26[2]	0.13[2]	0.24[2]		-0.06[n]	0.18[2]	0.16[2]	0.38[2]	0.30[2]	0.20[2]
**Adiponectin**	-0.16[2]	-0.18[2]	-0.06[n]	0.06[n]	0.17[2]	0.03[n]	-0.03[n]		0.09[1]	0.00[n]	-0.06[n]	-0.36[2]	-0.19[2]
**Adipsin**	0.18[2]	-0.00[n]	0.19[2]	-0.11[2]	0.20[2]	0.18[2]	0.14[2]	0.13[2]		0.06[n]	0.25[2]	0.15[2]	0.09[n]
**Follistatin**	0.05[n]	-0.02[n]	0.08[n]	-0.08[n]	0.14[2]	0.17[2]	0.20[2]	0.06[n]	0.04[n]		0.04[n]	0.04[n]	0.03[n]
**Leptin**	0.53[2]	0.12[2]	0.58[2]	-0.55[2]	0.12[2]	0.15[2]	0.35[2]	0.01[n]	0.25[2]	0.11[1]		0.72[2]	0.15[2]
**L/A ratio**	0.48[2]	0.16[2]	0.44[2]	-0.42[2]	0.00[n]	0.07[n]	0.30[2]	-0.50[2]	0.14[2]	0.05[n]	0.72[2]		0.22[2]
**MHR**	0.18[2]	0.11[2]	0.16[2]	-0.12[2]	-0.01[n]	0.16[2]	0.15[2]	-0.20[2]	0.11[2]	0.15[2]	0.15[2]	0.23[2]	

BMI, body mass index; WHR, waist-to-hip ratio; FM/WT, fat mass/weight ratio; SMM/WT, skeletal muscle mass/weight ratio; ECW/ICW, extracellular water / intracellular water ratio; L/A ratio, leptin/ adiponectin ratio; MHR, monocyte to high-density lipoprotein cholesterol ratio.

[1]p <.05; [2]p <.01; [n]p >.05
